# Cationic Surfactants: Self-Assembly, Structure-Activity Correlation and Their Biological Applications

**DOI:** 10.3390/ijms20225534

**Published:** 2019-11-06

**Authors:** Lucia Ya. Zakharova, Tatiana N. Pashirova, Slavomira Doktorovova, Ana R. Fernandes, Elena Sanchez-Lopez, Amélia M. Silva, Selma B. Souto, Eliana B. Souto

**Affiliations:** 1 Arbuzov Institute of Organic and Physical Chemistry, FRC Kazan Scientific Center, Russian Academy of Sciences, 8, ul. Arbuzov, Kazan 420088, Russia; luciaz@mail.ru (L.Y.Z.); tatyana_pashirova@mail.ru (T.N.P.); 2 Department of Organic Chemistry, Kazan State Technological University, ul. Karla Marksa 68, Kazan 420015, Russia; 3 Department of Pharmaceutical Technology, Faculty of Pharmacy, University of Coimbra (FFUC), Pólo das Ciências da Saúde, Azinhaga de Santa Comba, 3000-548 Coimbra, Portugal; s.doktorovova@hotmail.com (S.D.); anaritavfernandes@gmail.com (A.R.F.); esanchezlopez@ub.edu (E.S.-L.); 4 Department of Pharmacy, Pharmaceutical Technology and Physical Chemistry, Faculty of Pharmacy, University of Barcelona, 08028 Barcelona, Spain; 5 Institute of Nanoscience and Nanotechnology (IN2UB), University of Barcelona, 08028 Barcelona, Spain; 6 Networking Research Centre of Neurodegenerative Disease (CIBERNED), Instituto de Salud Juan Carlos III, 28702 Madrid, Spain; 7 Department of Biology and Environment, School of Life and Environmental Sciences, University of Trás-os-Montes and Alto Douro, 5000-801 Vila Real, Portugal; amsilva@utad.pt; 8 Centre for the Research and Technology of Agro-Environmental and Biological Sciences, University of Trás-os-Montes and Alto Douro, 5000-801 Vila Real, Portugal; 9 Department of Endocrinology of S. João Hospital, Alameda Prof. Hernâni Monteiro, 4200–319 Porto, Portugal; sbsouto.md@gmail.com; 10 CEB-Centre of Biological Engineering, University of Minho, Campus de Gualtar, 4710-057 Braga, Portugal

**Keywords:** cationic surfactants, 1,4-diazabicyclo[2.2.2]octane, self-assembly, solubilization, drug delivery systems, antimicrobial nanocarriers

## Abstract

The development of biotechnological protocols based on cationic surfactants is a modern trend focusing on the fabrication of antimicrobial and bioimaging agents, supramolecular catalysts, stabilizers of nanoparticles, and especially drug and gene nanocarriers. The main emphasis given to the design of novel ecologically friendly and biocompatible cationic surfactants makes it possible to avoid the drawbacks of nanoformulations preventing their entry to clinical trials. To solve the problem of toxicity various ways are proposed, including the use of mixed composition with nontoxic nonionic surfactants and/or hydrotropic agents, design of amphiphilic compounds bearing natural or cleavable fragments. Essential advantages of cationic surfactants are the structural diversity of their head groups allowing of chemical modification and introduction of desirable moiety to answer the green chemistry criteria. The latter can be exemplified by the design of novel families of ecological friendly cleavable surfactants, with improved biodegradability, amphiphiles with natural fragments, and geminis with low aggregation threshold. Importantly, the development of amphiphilic nanocarriers for drug delivery allows understanding the correlation between the chemical structure of surfactants, their aggregation behavior, and their functional activity. This review focuses on several aspects related to the synthesis of innovative cationic surfactants and their broad biological applications including antimicrobial activity, solubilization of hydrophobic drugs, complexation with DNA, and catalytic effect toward important biochemical reaction.

## 1. Introduction

Cationic surfactants are amphiphilic compounds, which can dissociate in water with the formation of surface-active cations. These molecules have the capacity of self-assembling [1], being widely used in biotechnology [2,3]. It can be expected that cationic charge of amphiphilic molecules should play an important role in biotechnological applications, determining the huge potential of cationic amphiphilic agents as drug carriers in pharmacy and biomedicine. Among them, those with a quaternary ammonium (QA) head group are of high relevance from the practical viewpoint with wide biotechnological applications [4].

Occurrence of positively charged fragments in amphiphilic scaffold is responsible for their attractiveness in nanotechnological applications as antimicrobial and bioimaging agents [5,6], corrosion inhibitors [7], supramolecular catalysts [8], stabilizers of nanoparticles and nanocarriers [9,10], and, especially as, drug and gene nanocarriers [11]. Cationic head groups of surfactants provide their high affinity toward biopolyanions, such as DNA [12,13], cell membranes, and intracellular organelles, such as mitochondrion, thereby initiating the development of perspective field of therapy, the so-called mitochondrion medicine [14,15]. Essential advantages of cationic surfactants are the diversity of their head groups allowing chemical modification and introduction of desirable moiety to answer the biotechnological criteria. The latter can be exemplified by the design of novel families of ecological friendly cleavable surfactants [16], with improved biodegradability, amphiphiles with natural fragments [17], and geminis with low aggregation threshold, including those bearing hydroxyl-groups capable of hydrogen bonding [18], among others.

Despite the fact that general positive trend occurs in the development of biotechnological protocols based on cationic surfactants including their use in the treatment of virulent diseases, the toxicity of cationic amphiphiles remains the main problem preventing their wide practical applications. Starting with the first and second generations of cationic charged liposomes [19], preclinical and clinical results testify the promising cationic liposomal therapy in the future [20]. In 2005, Medigene has developed paclitaxel encapsulated in positively charged lipid-based complexes for the potential treatment of cancer [21]. More recently, MediGene obtained positive results with EndoTAGTM-1 in a controlled phase II clinical trial in pancreatic cancer and triple receptor-negative breast cancer [22]. To date, few cationic agents and formulations are known to have application in ophthalmic practice [23,24,25]. However, the high effectiveness and potential applications of cationic surfactants continue to attract much attention of researchers, with main emphasis given to the design of novel ecologically friendly and biocompatible cationic surfactants. To solve the problem of toxicity, different ways are proposed, including the use of mixed composition with nontoxic nonionic surfactants and/or hydrotropic agents [26] in the design of amphiphilic compounds bearing natural fragments, some of which will be discussed herein.

A series of works focusing on the application of cationic surfactants in drug delivery have been recently reviewed [27], with emphasis given to their use for gene therapy [28,29,30,31], as a template for the synthesis of mesoporous materials [32], and also to their biocidal action against bacteria and fungi [33,34]. In this review, the fundamental approach based on structure-activity correlations between the chemical structure and self-assembly properties of amphiphilic compounds, in combination with their impact on functional activity of supramolecular systems, is taken as a basis for the analysis and recommendations made. The structure-activity correlation will be exemplified by the systematic study of series of mono-, di-, and tetra-cationic derivatives of 1,4-diazobicyclo[2.2.2]octane (DABCO) of different hydrophobicity and some amphiphilic molecules with natural fragments.

## 2. Structural Diversity of Biocompatible and Ecologically Friendly Cationic Surfactants 

One of the promising approaches in the design of building blocks for biomedical applications, which include formulations for drug delivery, is the creation of new multifunctional environmentally friendly, cleavable, green, and less toxic cationic surfactant systems answering the biotechnological criteria and improving biocompatibility, solubility, bioavailability, and biodegradability of therapeutics [35,36,37]. The promising strategy in this way is the design of cationic systems containing several covalently bound hydrophobic, hydrophilic groups, and various spacers to decrease surfactant concentration thresholds. In this review, we have therefore started from the so-called gemini surfactants demonstrating low aggregation threshold and morphological lability [38,39]. To promote surfactant biodegradation and ecological safety, strategies for preparation of surfactant with hydrolysable ester bonds and cleavable amide links were employed. The new class of biocompatible surfactants with polar head groups containing natural moiety such as sugar-, peptide-, and pyrimidine-bearing moieties are promising drug and gene carriers for intracellular delivery [40]. Importantly, they provide a widened spectrum of intermolecular interaction (hydrogen bonding, electrostatic, and van der Waals), stacking and complementary interactions upon self-assembly, and binding the drug, thereby combining high efficacy with lower toxicity [41,42]. 

### 2.1. Gemini Surfactants

In recent years, particular attention is focused on gemini surfactants due to their advanced structure-activity correlation, superior aggregation capacity, and versatile morphological behavior [4,43]. Unlike conventional single head surfactants, gemini analogs consist of two equal head groups bearing two tails bridged with a spacer. Typically, the ammonium is substituted by two methyl groups or short alkyl chain group and a long aliphatic alkyl chain, which contributes with its lipophilicity to the overall amphiphilic character of the molecule. The length and nature (flexibility and hydrophilicity) of the spacer may serve as a tool for the tuning of their size, shape, and morphology, including sphere-to-rod or micelle-to-vesicle structural transitions [44,45]. Alkyl chains of various lengths (C2 to C10) are most frequently used, being the properties of the gemini surfactant in question influenced by the fact if odd or even number of ethylene groups are used [46]. 

**Figure 1:**
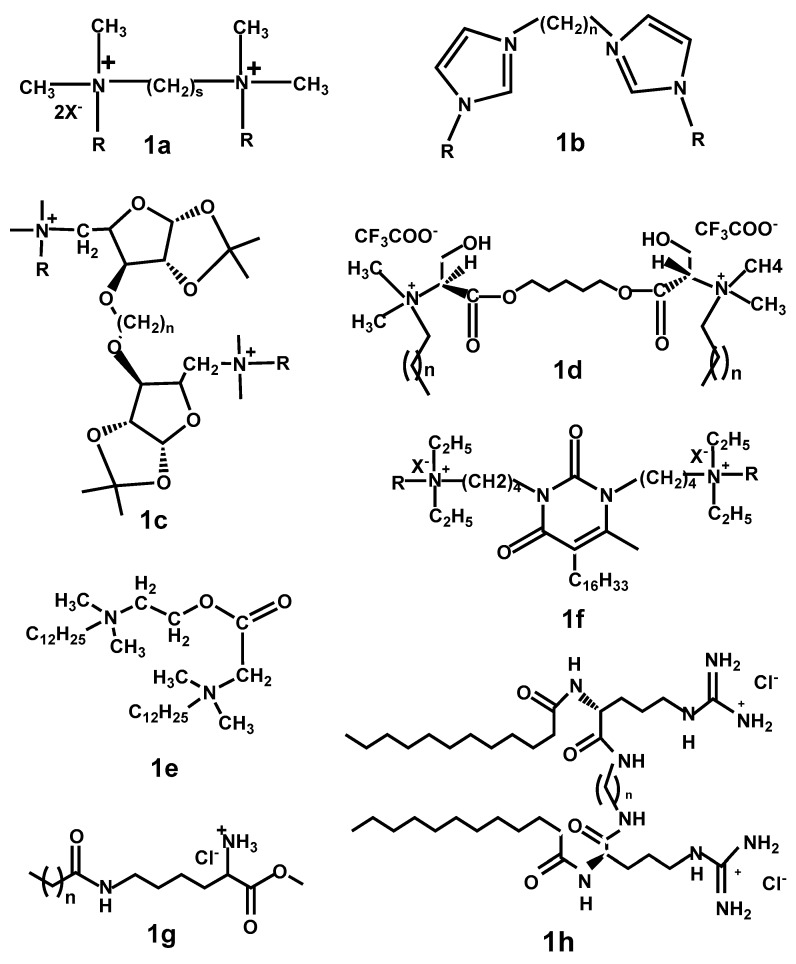
Examples of cationic quaternary ammonium surfactants, **1a:** R = C_n_H_n+1_, s = 4; **1b:** R = C_n_H_n+1_, *n* = 2,4; **1c**: R = C_12_H_25_, C_16_H_37_, C_18_H_37_, *n* = 3; **1d**: *n* = 10; **1f:** R = C_10_H_21_, X^−^ = Br^−^; **1g**: *n* = 10, 12, 14; and **1h**: *n* = 6, 9, 12.

The simplest QA gemini surfactants (**1a**) are based on two molecules of the prototype quaternary ammonium surfactant (QAS), cetyl trimethyl ammonium bromide (CTAB), or dodecyl trimethyl ammonium bromide (DTAB), linked by an alkyl spacer at the ammonium group. Examples of such structures are presented in Figure 1. The structure is usually annotated as *m-s-m*, where *m* and *s* represent the number of carbon atoms in alkyl chains and in polymethylene spacers, respectively. The quaternary ammonium group can also be contained in an imidazole heterocycle (**1b**). In this case, a long alkyl chain providing lipophilicity is linked to 3’ nitrogen in the imidazole ring, and a spacer links 1’ quaternary ammoniums. 

Alternatively, cationic QA gemini surfactants may also contain a natural moiety [47,48,49]). Examples are: (i) glucose moieties containing gemini surfactant (**1c**), where the spacer links the monomers at hydroxyl group of glucose moiety [49], as shown in Figure 1 and (ii) serine-based cationic gemini surfactant (**1d**), where the spacer links two serine nitrogen atoms, quaternarized by substitution with a long alkyl chain [48], Figure 1.

Due to their enhanced hydrophobicity, gemini surfactants demonstrate much lower critical micelle concentration (CMC), as compared to conventional surfactants. Conductomery, tensiometry, potentiometry, and other data indicate that a decrease in CMC by about two orders of magnitude occurs on transiting from monocationic to gemini surfactants with the same alkyl tail [50]. In the case of dicationic surfactants with hydroxyethyl fragment in the head group, an additional five-fold decrease in the CMC value is observed [51]. Another important feature of gemini surfactants is their enhanced solubilization capacity, exemplified by solubility of *p*-nitrophenyl esters of carbonic acid in mono and dicationic surfactant solutions, as compared to water [52].

The introduction of a labile group, typically an ester group, results in production of an easily cleavable molecule, which degradation would begin exactly at the ester group. The ester group can be introduced between quaternary ammonium head and alkyl tail (**1e**) (Figure 1) [53], or in the spacer [54]. The former type falls in the group of esters quat surfactants, which are cleaved at the ester groups forming much less active products, offering, thus, a biodegradable and environmentally friendly class of surfactants. The latter group degrades into two QA molecules differing in hydroxyethyl- or hydroxymethyl-chain, produced by the cleavage of the asymmetric spacer. The resulting products maintain surface activity, but this is greatly inferior compared to the original gemini [54].

### 2.2. Ionic Liquids 

Other types of ecologically friendly amphiphiles that have gained much interest are the ionic liquids (IL), especially those bearing methylimidazolium (mim) cation. Dicationic imidazolium surfactants were reported to be used as supramolecular models of anionic abiotic receptors and exhibit high binding activity to anionic drugs exemplified by valproate [55]. 

Lauryl isoquinolinium bromide, studied by Zhang et al. [56], was compared with homologs of different hydrophobicities and with typical surfactant lauryl pyridinium bromide. The CMC value evaluated was treated in term of π-π stacking effect contributed by aromatic rings. Rheological behavior of IL C_14_mimBr in aqueous solution has been evaluated in the presence of sodium salicylate [57]. Maximal viscoelastic properties are found to correspond to the 1:0.6 surfactant/salt molar ratio and to be contributed by ion-pairing between them. Novel class of IL with short alkyl tails and long chain counter-ions that form catanionic surfactants have been designed. Three ionic liquids based on the imidazolium cation with rather short alky tails (*n* ≤ 8) and ibuprofenate as counterion have been synthesized [58]. The morphology and composition of aggregates are strongly controlled by alkyl chain length associated with imidazolium cation. Under the dilution regime, the micelle-to-vesicle transition can occur. In an analogous manner, salt-free catanionic IL based on alkylated mim as cationic head (*n* varied from 6 to 10) and alkyl sulfates as counterions were prepared [59]. Amphiphilic character of IL synthesized can be tailored by the hydrophobicity of both cationic group and counterion, with CMC markedly decreasing with an increase in alkyl chain length. 

Hydrophobized imidazolium salts studied are capable of self-assembling with the formation of vesicles without any additives or impulses [60]. This has been attributed to the specific packing parameter of the molecules. Mester et al. [61] addressed the toxicity of the mim-based IL with the role of counterion emphasized and reported that the chaotropicity of the anion is shown to be a key factor controlling the toxicity of IL studied. The genotoxicity of nanoparticles decorated with the imidazolium salts was lower compared with those covered with other cationic surfactants [62,63]. A weak point for IL is the fact they pose several risks to human health and environment; thus, the positive impact of IL should be evaluated with due caution.

### 2.3. Cationic QA Surfactants Containing a Natural Moiety 

In an effort to obtain quaternary ammonium compounds (QASs) that would be less toxic than existing cationic surfactants, novel quaternary ammonium compounds (QACs) that contain a natural moiety have been synthetized by various research groups. The natural moieties include nucleotides/nucleosides (nucleolipids), amino acids (lipoaminoacids), peptides, and diterpenoids. By making use of naturally occurring materials, it is believed that the resulting novel cationic surfactants provide better biocompatibility and limited toxicity and improve the environmental safety profile. Many of these compounds contain a cleavable group in order to provide a compound that decomposes easily to less active or inactive molecules. These cleavable compounds fulfil the prerequisites of biodegradable, environmentally friendly surfactants.

#### 2.3.1. Pyrimidinophanes: Macrocycles with Nucleotide/Nucleoside Moiety

Special class of hybrid amphiphiles involves the so-called nucleolipids that are composed of hydrophobic tails covalently bound to nucleotide or nucleoside moieties. A wide series of novel macocyclic, dicationic amphiphiles bearing pyrimidinic fragment (**1f**) (Figure 1), in particular, uracil, have been studied. An excellent review of pyrimidinophanes structure, synthesis, and application by V.E. Semenov is recommended for further, detailed reading [64].

Acyclic and macrocyclic pyrimidinophane analogs with varied size of macrocycle, alkyl chain length, and counter-ion nature were compared. Kharmalov et al. [65] studied aggregation behavior of a pyrimidinocyclophane with an uracil moiety. This pyrimidinocyclophane has cationic character at pH = 1 and is insoluble in water at pH < 4 were it forms cationic aggregates. An unusual structural transition has been revealed from large aggregates to lower micelle-like assemblies with an increase in amphiphile concentration, reversibly soluble upon increase of environment pH. The studied pyrimidinocyclophane forms, in excess with Triton X-100, stable, normal micelle aggregates in alkaline and acidic conditions [65]. In a series of macrocyclic uracilophanes with different spacer rigidity, it was shown that their aggregation behavior is controlled by the molecule geometry; the higher rigidity, the lower aggregation capacity, shown by the difference in CMC from 1 mM for “monomeric” and multiuracilophane, to 10 mM for cryptand-like uracilophane [66]. Macrocyclic scaffold markedly increased the packing parameter up to the value of 1, thereby tuning the model of association and morphological behavior of uracilophanes compared to conventional surfactants [67]. It was shown that CMC values of pyrimidinic surfactants with ammonium head groups are lower as compared to trimethylammonium counterparts of the same hydrophobicity, with CMC decreasing upon the transition from acyclic to macrocyclic compounds, replacement of bromide by tosylate counter-ion, and especially with the introduction of long alkyl chain into the pyrimidine fragment [66]. The specificity of self-assembly is clearly demonstrated on the example of sulfur-containing pyrimidinophane, for which temperature and concentration dependent gelation occurs [68]. The potential of these surfactants is in formation of self-assembled soft matter with negatively charged matter of interest, e.g., nucleic acids or proteins, with potential to form stable aggregates with capacity to control their release and facilitate their intracellular delivery.

Pyrimidinic amphiphiles cause decrease of the pH of the solution with increasing concentration [66]. This phenomenon has been attributed to shielding effect preventing the access of counter-ion to the head groups. As a result, high uncompensated charge is accumulated on the surface of aggregates that cause the polarization up to dissociation of water molecules in solvate shells of head groups. Generated hydroxide ions bind with cationic head groups, while unpaired hydroxonium ions acidify the bulk solution [66]. This effect is of importance from the viewpoint of practical application of these surfactants, since it opens the possibility to design the pH controlling nanocarriers bearing uracilic moieties. Indeed, for these compounds, stimuli responsive aggregates are obtained that demonstrate diverse structural behavior and controlled uptake and release of hydrophobic guest and characterized of low toxicity [69].

#### 2.3.2. Lipoaminoacids

In an effort to limit environmental burden and toxicity, synthesis of amino acid-residue containing QACs has been proposed. The structure varies from monomeric, alkylated amino acids, to gemini QACs [53,70]. Alkylated amino acids comprise a hydrocarbon tail linked either via lysine α-amino group or via ε-amino group. Perez et al. observed that QACs belonging to N^ε^-acyl-lysine generally showed decreased hemolytic activity in comparison to N^α^-acyl lysine QACs (**1g**) (Figure 1) [71]. Increasing chain length of the alkyl tail resulted in compounds with higher hydrophobic component and subsequently lowers the CMC [71]. 

Gemini lipoaminoacids comprise two amino acid residues linked by a spacer either via amino acid carboxyl group [72] or via N^α^ quaternary ammonium [53]. Gemini analogs functionalized with amino acid moieties demonstrate superior surface activity, and some derivatives promote transfection efficacy upon their conjugation with liposomes [48]. Alkylated lauryl arginine monomer and gemini with short (C6) spacer assemble into micelles; compounds with longer spacer (C9, C12) form vesicles. Diacylglycerol arginine derivatives also showed capacity to form vesicles on their own or integrate liposomes consisting of phospholipids. Examples of amino-acid residue containing QACs are given in Table 1.

Most of lipoaminoacids show mild antibacterial activity, serine-based lipoaminoacids were successfully tested for DNA complexation. Lysine-based and serine-based lipoaminoacids also showed capacity to enhance skin penetration of model drugs encapsulated in liposomes containing these compounds [73,74]. Despite these interesting properties, most lipoaminoacids maintain the issue of interaction with cell membranes, leading to hemolysis, frequently already at doses required for inhibition of bacterial growth. This limits the use of lipoaminoacids to topical use, still making them a promising group of novel disinfectant agents or skin permeation enhancers. As evidenced by Tavano et al. [72], incorporation of further lipid component, namely cholesterol (Chol) or phospholipids, can limit the hemolytic activity of arginine-based QACs (**1h**) (Figure 1) while the antimicrobial activity is preserved; making this class of surfactants active against bacteria at concentrations below those at which hemolysis occurs. Further insight on lipoaminoacids, not limited to cationic amphiphiles, can be found in work by Bordes and Holmberg [74].

#### 2.3.3. Other QASc Containing Natural Moiety: Peptides, Diterpenoids 

Surfactant-like pseudopeptides comprise few hydrophilic head groups originating in amino acid residues and a hydrophobic tail. Compounds of this structure have capacity to self-assemble into various colloidal structures, including vesicles, nanotubes, micelles, or nanofibres. Synthesis of pseudopeptidic cationic amphiphiles has been reported, whose admixtures with anionic component form supramolecular biocompatible thermogels with potential applications in cosmetic and pharmaceutical products [79]. 

Novel amphiphilic diterpenoid derivatives have been synthesized showing structural behavior, capacity of integrating with lipid bilayer, and binding with oligonucleotide have to be solely controlled by the nature of the counter-ion, i.e., bromide or tosylate ion. While tosylate derivative displayed typical behavior and formed micelle-like aggregates, bromide analog underwent structural transitions at a certain concentration. Importantly, only the bromide derivative, one of two, could penetrate through the membranes. Much attention has been paid to the biocompatible and ecologically safe hydrogels that are assumed to result from synergetic contribution of hydrophobic effect, hydrogen bonding, and π-stacking interactions [80]. 

## 3. Self-Assembling Strategies for Construction of Soft Nanomaterials for Biomedical Application

Soft materials are widely used for the modification of drug delivery and targeting [81,82]. First of all, these systems can be used for the development of aqueous formulations loaded with poorly-soluble drugs. Many different drug delivery systems have already been tested, e.g., nanoemulsions, hydrogels, microspheres, liposomes, micelles, nanoparticles, and nanocrystals. Recently, many efforts have been taken for the development of biocompatible nanocarriers with colloid phase for hydrophobic molecules [83], which allow for balancing between advanced efficacy and low toxicity. The ultimate aim is to address the demand of pharmaceutical industry, which require novel formulations with multiple functionality and diverse types of carriers for controlled binding/release of drugs, minimization of biodegradation and side effects, and improvement of bioavailability [84]. 

QAC form colloidal aggregates of interest for pharmaceutical industry, applicable for delivery of small molecule active ingredients, peptides, and nucleic acids. Micelles and vesicles formed by QAS alone or in combination with neutral or oppositely charges surfactants are of interest for their increased solubilization capacity, especially for hydrophobic drugs (see further). Cationic stabilizing agents are also of crucial important for lipid based colloidal carriers, where they act as stabilizers and provide positive overall surface charge. Furthermore, inorganic nanomaterial synthesis often relies on use of cationic surfactants as templates.

Analysis of recent literature testifies that due to their structural diversity cationic surfactants tend to form different morphological systems from micelles to vesicles, with the stimuli responsibility observed [85]. This is especially manifested in case of cationic geminis, for which size and morphology strongly controlled by structural characteristics, as well as catanionic surfactants demonstrating unique morphological and rheological behavior [27]. These properties are responsible for the versatile functionality of cationic surfactants and their wide application in modern nanotechnologies as modifiers of soft multifunctional nanocontainers (liposomes, nanoemulsions, and solid lipid nanoparticles) and metal nanoparticles improving their stability and functional activity [10,86,87]. In this review, we emphasized the advantages of cationic surfactants as nanocarrier modifiers allowing to (i) increase their colloidal stability; (ii) improve the encapsulation efficacy and loading capacity; (iii) increase the affinity toward both therapeutic loads and cell membranes; (iv) improve the penetrating through biological barriers, to provide conditions for multidrug delivery; and (v) extend markedly the spectrum of their activity due to introducing additional functionality, e.g., antimicrobial properties, gel-like or liquid crystalline behavior, and catalytic effect. Meanwhile, one of the key challenging tasks in these fields is to achieve a delicate balance between the advanced functional characteristics of modified nanosystems and possibly unfavorable changes in toxic properties. These points were illustrated by the widely used nanocarriers, such as liposomes, nanoemulsions, and metal ion nanoparticles.

### 3.1. Aggregates of Oppositely Charged Surfactants: Catanionic Systems

The use of binary drug delivery systems based on cationic/anionic surfactant mixtures offers many advantages [27,88]. These systems are characterized by a diverse morphological behavior and demonstrate a high synergetic effect due to the strong negative interaction parameter β. The so-called catanionic vesicles are spontaneously formed therein under non-stoichiometric ratio, which is governed by both favorable enthalpy changes contributed by high electrostatic attraction between head groups and an increase in entropy contributed by release of counterions. Catanionic vesicles are superior over typical lipid-based liposomes, since they need no extrusion or sonication treatment; show long-term stability and reduced cytotoxicity compared to single constituents; and can be easily modified in their size and charge character. Besides structural diversity, gel-like and viscoelastic behavior can be easily attained in catanionic systems [89,90].

Electrostatic interactions between cationic and anionic surfactants yield amphiphilic ionic pairs that may form aggregates of different morphologies with varied shape and size, e.g., spherical and rod-like micelles, disk-shaped aggregates, and vesicles [91,92]. Tests on cytotoxicity confirmed that catanionic mixed compositions could more effectively cell uptake of cancer cells [82]. Catanionic vesicles based on anionic surfactant sodium dodecyl sulfate (SDS) and cationic surfactant CTAB have been obtained and used for insulin delivery [93]. Spontaneously formed catanionic vesicles based on lactose can upload the hydrophobic phthalocyanine and improve antitumor effect [94]. Simple design of the light-sensitive vesicles, commercially available and relatively inexpensive, can be used as a new class of rheological fluids with the light-controlled properties [95].

### 3.2. QASs in Role of Stabilizing Agents

#### 3.2.1. Cationic Liposomes

Liposomes are drug carriers with the large-scale application, with the cationic liposomes widely used as transfection agents. It was documented that liposomes bearing positive surface charge were selectively cumulated in endothelial tumor cells. In the case of cationic niosomes, their uptake by the cells in rat retina and brain was more effective and selective as compared to their uncharged counterparts [96]. Multifunctional cationic liposomes based on gemini surfactants and a helper lipid dioleoyl phosphatidylethanolamine (DOPE) were successfully used for the co-delivery of RNA and doxorubicin into cells [97]. Liposomes fabricated on the basis of diacyl glycerol-arginine surfactant and a helper lipid dipalmitoylphosphatidylcholine (DPPC) loaded with ciprofloxacin and 5-fluorouracil show antibacterial activity, and therefore these formulations were supposed as the dual-purpose preparations [70]. Liposomes modified with cationic double-chained surfactant didodecyldimethylammonium bromide (DDAB), Chol, and non-ionic surfactants were fabricated for delivery of water-insoluble drug curcumin, which improved cell uptake of the carriers compared to DDAB-free formulations [98]. 

It is known that liposomes admixed with cationic surfactants demonstrate improved transdermal delivery of some drugs. Negative charge of skin surface favors the penetration of positively charged carriers thereby facilitating the transdermal drug transport. This is exemplified by the data on improved transdermal delivery of drug meloxicam by the modification of liposomes with cationic surfactant cetylpyridinium chloride [99]. Nogueira et al. described a formulation based on novel biocompatible amino acid-based surfactants (natural cationic lipids) that form vesicles, with their cytotoxicity controlled by the structure of surfactants [75,100].

#### 3.2.2. Nanoemulsions

One of the most common drug delivery systems that has found numerous practical applications are the nanoemulsions. Usually, these systems stabilized with synthetic surfactants. For this purpose, novel promising cationic diamidequat-type surfactants are designed with improved biocompatibility [101], accomplishing the demands for pharmaceutical and cosmetic use. Bionanoemulsions produced with the help of cationic and non-ionic poloxamers were used for improving the antioxidant activity of natural phenolic antioxidant curcumin [102]. Block ionomer complexes formed by poly (ethylene glycol)-block-poly(4-vinylbenzylphosphonate) (PEG-b-PVBP) and cationic surfactants was used for encapsulation of doxorubicin and its controlled release [103]. Spontaneously self-assembled nanosized particles are stable over wide variety of experimental conditions and highly sensitive to the structure of cationic surfactants. Modification of polymer matrix of contact lenses with cationic surfactants (benzalkonium chloride, cetalkonium chloride), proposed by Bengani et al. [104], allows to prolong therapeutic modality of ophthalmic drug dexamethasone 21-disodium phosphate adsorbed at modified surfaces. 

### 3.3. QASs in Role of Inorganic Nanomaterial Synthesis 

Numerous promising nanomedicine applications involve metal nanoparticles (NP), therefore the synthesis, functionalization, and stabilization of NP is an important task. Cationic surfactants are widely used in synthetic protocols, surface decoration, and stabilization of NP [25,105]. Typically, reverse microemulsions are used as nanoreactors allowing for the fabrication of particles with controlled morphology and size and preventing their undesirable agglomeration. The composition of microemulsion, especially the nature and concentration of surfactants markedly affect the properties of NP fabricated. This is exemplified by the synthesis of silver nanoparticles in reverse microemulsion stabilized with surfactants of different types. The charge character is shown to control the size of NP, with admixture of cationic surfactant exhibiting a stabilizing effect. Much attention has been paid to the structure-activity correlation [44], which makes it possible to optimize synthetic protocols and modify the properties of NP. Imidazolium gemini surfactant capped silver NP have been synthesized by Datta et al. [44]. Spacer length is reported to control the morphology of NP, which varied from elongated nanostructures in the case of short spacer composed of two methylene groups, to spherical one in the case of longer spacers (five to twelve methylene units). Importantly, not only the structure but also the orientation of surfactant molecules may affect the properties and functionality of NP [106]. Imidazolium gemini surfactant coated gold NP were efficient in piroxicam-loading and sustained its release, as the gemini surfactant formed a bilayer on NP surface, providing suitable environment for drug loading while not exceeding the cytotoxicity observed when using different cationic surfactants [107]. Synthesis of mesoporous silica NP by Stober method relies on hydrolysis of organoalkoxysilane in presence of CTAB. More details into silica NP synthesis methods and used can be found elsewhere [108,109].

## 4. Quaternary Ammonium Surfactants in Pharmaceutical Applications

Several current and prospective pharmaceutical dosage forms gain considerable advantage over neutral or negatively charged drug delivery systems when they present overall cationic charge. This is the case of colloidal systems aimed for ocular drug delivery, where positively charged aggregates interact with negatively charged ocular surface and hut provide longer contact time, resulting in improved ocular bioavailability of the administered drug and provide a base of long-time, prolonged release of the drug [24]. An improvement of skin penetration was reported after application of cationic amphiphiles, mainly amino-acid based [73,76,78,99]. Most importantly, cationic amphiphiles improve drug loading of many actives into pre-existing drug delivery systems (liposomes, hydrogels, and lipid particles) or improve substantially their solubility by complexing the drugs into self-assembled colloidal aggregates, micelles or vesicles. 

### 4.1. Delivery of Small Drug Molecules 

QAC often show superior drug solubilization efficacy than neutral materials. In part, this is due to amphiphilic character of QACs, frequently beneficial for formulation of hydrophobic drugs. Improved solubilization capacity then results in higher encapsulation efficacy of drug delivery system in question. Furthermore, inclusion of cationic amphiphiles has impact on drug release.

Smart delivery systems based on cationic surfactants have been designed and used for controlled binding/release of antibiotics, doxycycline and oxytetracycline [110]. The pH sensitive drugs can be selectively bound within nonpolar core (zwitterionic form of drug) or in cationic shell (anionic) form depending on the pH of the solution, while the release of drug was magnesium driven process.

#### 4.1.1. Factors Determining the Solubilization Efficacy

Efficacy of micellar solubilization depends on numbers of factors, such as structure of surfactants, aggregation number, morphology of aggregates, ionic force, temperature, as well as on the structure of the solute. An increase in the alkyl chain length of cationic surfactants with trimethyl ammonium head group is shown to result in the linear increase in the solubility of hydrophobic dyes. This may be explained by an increase in nonpolar core of surfactant aggregates. Gemini surfactants demonstrate enhanced solubilizing properties as compared to monomer analogs. This is probably promoted by the lower CMC of gemini surfactants. The nature of counter-ion slightly influences the solubilization capacity of aggregates. On the other hand, the nature of head groups (e.g., non-ionic versus ionic and imidazolium versus trimethyl ammonium) has been reported to markedly affect the solubilization efficacy of surfactants [111,112]; in particular the ability of interactions between the head groups and solutes strongly facilitates the solubilization power. This means that binding of the probe occurs not only within the nonpolar core but also by the periphery, as for example in the case of non-ionic surfactants that have polyoxyethylene mantle [111]. On the other hand, the study of cationic systems testifies the existence of cation-π as well as π-π interactions between drugs and surfactant (dopamine hydrochloride or and acetylcholine chloride and 1-tetradecyl-3-methylimidazolium bromide) [112]. 

Among ionic surfactants, the cationic surfactants show superior solubilization capacity toward polyaromatic hydrocarbons, such as anthracene and pyrene, which is due to the favorable electrostatic interactions between the positively charged head groups of amphiphiles and electron donating aromatic rings [113]. The use of mixed systems based on gemini and conventional surfactants makes it possible to stabilize micelles and enhance the solubility of poly polycyclic aromatic hydrocarbons (naphthalene, anthracene, and pyrene) [114]. The solubilization study of naphthalene and stearic acid demonstrated that trimeric surfactant presented better potential as solubilizer compared to typical monomeric and dimeric surfactants [115].

#### 4.1.2. Drug-Amphiphile Interactions

Amphiphilic molecules and drugs can form mixed assemblies that result in the increase in the solubility, stability, and bioavailability of drugs and concurrently, in changing in the micellization process [116]. Different morphology of resulting mixed systems can be used for controlled release of loads for the derivatives of cephalosporin, the mutual influence of surfactant and drug is evaluated, in particular, the CMC of cationic surfactants are shown to increase with the drug added [117]. The influence of cationic surfactant cetyltrimethylammonium bromide on the spectral characteristics and acid-base properties of the drugs irbesartan, losartan, and valsartan has been recently demonstrated. The interaction of losartan with CTAB is followed by an increase in the absorbance of the drug and shift of pKa. This specific interaction allows for sensitive quantification of drugs in complex pharmaceutical formulations [118]. 

Amphiphilic surfactants have also been used for formulating amphiphilic drugs and, in this case, the probability of producing mixed assemblies markedly increases. For the system based on amphiphilic drug promethazine and cationic imidazolium surfactants thermodynamic parameters have been estimated which testified that synergetic effect occurred, thereby supporting the formation of mixed aggregates with higher stability and more compact packing of monomers. Along with electrostatic interactions, the contribution of hydrophobic effect has been emphasized, which was exemplified by the imidazolium surfactant-ibuprofen system [119]. The structure of aggregates of ionic liquids based on imidazolium salts and surface-active drug (e.g., ibuprofen) may be controlled by the variation of alkyl chain length of cationic surfactant [58]. In a mixture of ibuprofen and cationic surfactant with short tail, spherical micelles are reported to be formed. An increase in the alkyl chain length of imidazolium surfactant results in an increase in the size of micelles. Meanwhile, within the low concentration range the formation of vesicles has been observed. The development of a new strategy for the fabrication of double-wall nanotubes of different morphology by the mixing of two oppositely charged antitumor drugs has been reported [120]. Preliminary animal testing showed that these nanotubes markedly prolong the contact of drug with tumor cells. 

### 4.2. Protein and Peptide Delivery and Peptides for Drug Delivery

#### 4.2.1. Protein and Peptide Delivery by Cationic Surfactants

With respect to protein-surfactant interactions several factors should be taken into account, e.g., the charge of head group, the length of hydrophobic fragment, and the conformation of protein. Conformation changes of the proteins upon their binding with surfactants influence the functional activity of the proteins and provide the molecular level information on the protein-surfactant interactions. The binding interactions of the surfactants with bovine serum albumin (BSA) were exemplified by computer simulation the results disclosed that the surfactants bound stably between hydrophobic subdomain IIA and IIIA where tryptophan-213 residue [121]. In the case of gemini with pyridinium group, conformation changes are due to the π-stacking interactions of pyridinium ring of the surfactant and pyrrole ring of tryptophan residue of BSA [122]. The role of micellar medium is equally important in drug-protein the interactions [123]. The binding strength of drug diclofenac with BSA increases if the preparation is used in micellar formulation based on cationic surfactant. 

#### 4.2.2. Drug Delivery by Quaternary Ammonium Containing Peptides

The powerful tool for the intracellular delivery of different therapeutic molecules is the conjugation of therapeutic cargo with the cell penetrating peptides (CPP). They are regarded as promising transporters for a variety of biomolecules with low bioavailability across the lipid bilayer. Positively charged arginine-rich cell penetration peptides were successfully used as vectors for the intracellular transport of small bioactive molecules involving endocytosis as a key cellular uptake pathway [124]. An effective strategy for improving the cellular uptake consists in attachment of short hydrophobic fragment, the so-called penetration-accelerating sequence to arginine-rich CPP. The important role of the hydrophobicity of the CPP-conjugated formulation was particularly emphasized [124]. Ye et al. developed a simple method for the intracellular delivery of negatively charged phosphopeptides through their non-covalent conjugation with cationic vector based on amphiphilic tripodal peptide analogues [125]. 

An effective peptide-based strategy was developed by Cheetham et al. [126], in which amphiphilic drug molecules were designed through conjugation of hydrophobic drug camptothecin with Tau-β-sheet-forming peptide. Resulting nanostructures are reported to be characterized by high loading capacity and controlled drug release, which allows this protocol to be extended for delivery of related drugs. Improved mitochondrial targeting behavior was achieved in the study [127], which reported on the dual conjugation strategy consisting in the combination within a molecule of both CPP and a targeting agent. These conjugated carriers show superior intracellular delivery of therapeutic unit with low penetrating ability across the membrane. Meanwhile the use of single transporter molecules, CPP, or mitochondrial targeting sequence appeared to be less effective.

### 4.3. Gene Delivery 

Nucleic acid molecule typically cannot enter the cell on its own. Currently, it is recognized that a successful non-viral, intracellular nucleic acid delivery system is dependent on a cationic component (e.g., [128]). This is responsible for DNA/RNA condensation and packing, and for initial contact of gene delivery system with cell surface, which has overall negative charge, originating from phosphate groups of various phosphatidylcholines. Cationic agents (polymers or lipids) show high affinity for the phosphate backbones of DNA. In order to condense and deliver nucleic acids effectively, lipoplexes typically require presence of a helper lipid in the formulation. As helper lipid, DOPE is used more frequently, and is included in various commercial transfection reagents. Dimyristoylphosphatidylcholine (DMPC) or Chol can also act as helper lipids. Despite availability of several cationic lipid-based transfection reagents, none of it is suitable for clinical use due to its toxicity (mainly hemolysis and potential of trigger of immune system reactions). Therefore, synthesis of new cationic agents with increased DNA binding efficiency and decreased toxicity potential is a lively field with possible great impact on translation of gene delivery from non-clinical to clinical applications.

Efforts have been made to design novel QAS for gene delivery that would be able to deliver nucleic acids without complementary action of helper lipids. Inspired by rigid, planar structure of Chol, Andrzejewska et al. synthetized cationic gemini surfactants that comprise a rigid cyclic moiety and condensed DNA of various length into stable aggregates, being the surfactant with longer spacer slightly more efficient in DNA binding [129].

Cardoso et al. reported that 12-5-12 and 12-10-12 QAS complexed DNA successfully but their transfection efficiency was low, even in the presence of helper lipids, and toxicity towards cells was observed [130]. As an alternative, serine based cationic gemini surfactants series with different alkyl chain length and amine, amid, or ester groups linking the serine moieties with the spacer, were successful in DNA binding and showed efficient in vitro transfection, without marked decreased of cell viability. The nature of linkage between head-group and spacer influenced markedly DNA release, being the ester bond containing surfactants the least prone to release DNA upon contact with vesicles mimicking cell membrane [48].

Cationic amphiphiles bearing pyrimidine fragment have been explored with the aim of highlighting the role of complementary interactions involving pyrimidine moiety of amphiphiles and nucleotide bases of DNA. Among them bola-form, single-head, and dicationic pyrimidinic surfactants were used, which enable us to differentiate the contribution of electrostatic and hydrophobic interactions to the DNA-surfactant complexation [131]. It has been shown that dimeric pyrimidinic gemini with enhanced hydrophobicity and high micellization capacity demonstrated highest condensing activity exceeding that of conventional *m-s-m* geminis and bola-form analogs. It was shown that complexation activity of surfactants toward oligonucleotide increases from ammonium surfactants < phosphonium surfactants < 1,4-diazabicyclo[2.2.2]octane based surfactants [132]. Gemini pyrimidine surfactant (SPYRIT 68) showed better in vitro transfection efficiency than monomer cationic lipid (SPYRIT 7), but also higher cytotoxicity. A combination of both cationic lipid and gemini surfactant showed even better transfection efficiency and decreased cytotoxicity. A possible mechanism of action was the induction of endolysosomal membrane rupture, induce by gemini SPYRIT 68, documented by diffuse intracellular distribution of Rab-7, a Rho-GTPase present on late endosomal membrane [133].

In a series of hydroxyethylated analogs of *m-s-m* geminis, i.e., 16-*s*-16 (OH), with *m* varying between four, six, and 12, the spacer length markedly influenced the transfection efficacy. The maximum transfection was observed in the case of the longest spacer [134].

### 4.4. Antimicrobial Effects

Due to the ability of interaction with cell membranes, namely attraction by overall negative charge of the membrane and insertion, cationic amphiphiles facilitate contact with cell surfaces and intracellular delivery of the cargo. Frequently, the interaction of cationic amphiphiles leads to perturbation of membrane, and, ultimately, may lead to membrane rupture. Disruption of cell membrane integrity by cationic surfactants seems to affect both prokaryotic and eukaryotic cells. Hemolysis is therefore often used as proof of cationic amphiphile interaction with eukaryotic membrane [72,76], but in practical terms it excludes many interesting cationic amphiphiles for the use for which they were designed. Apart from cell membrane damage, CTAB is known inductor of oxidative stress, even when incorporated in lipid nanoparticle systems that shield partly its effects on cell [105].

Combination of cationic amphiphiles with helper material might shield their toxic effects towards cell membranes, but this may not be sufficient for clinical use. Therefore, use of cationic amphiphiles is often limited to local administration, where they already showed interesting properties, namely in ophthalmic applications and for skin penetration enhancements. Furthermore, antimicrobial activity that cationic amphiphiles exert makes them suitable candidates for topical disinfectants and antiseptic agents. Tavano et al. reported that a series of arginine-based cationic gemini surfactants, incorporated in liposomes, showed antimicrobial efficiency (represented by inhibition concentration for 50% of bacteria, IC50) at concentrations lower than those at which hemolysis occurs [72]. Dialkylamino and nitrogen heterocyclic analogues of hexadecylphosphocholine and CTAB showed strong cytotoxic activity against several cancer cell lines, fungi (antifungal activity) and protozoans (antiprotozoan activity), all at concentrations much inferior to those at which hemolysis was observed, being the most active compound, in terms of cytotoxicity, a dibutylamino analogue of CTAB [135].

Colloidal complexes of cationic surfactants with negatively-charged hyaluronic acid (HyA) were found to reduce the cytotoxicity induced by surfactants. the specific sensitivity of different cell types to surfactant treatment was determined [136]. Dequalinium is another example of a QAC that despite interesting antimicrobial properties cannot be used for systemic infection treatments but found application in treatment of topical infections. Dequalinium forms vesicles, DQAsomes, which allow for encapsulation of further antimicrobial agents [137].

## 5. Self-Assembled Quaternized Derivatives of 1,4-Diazabicyclo[2.2.2]Octane and Quinuclidine

**Figure 2:**
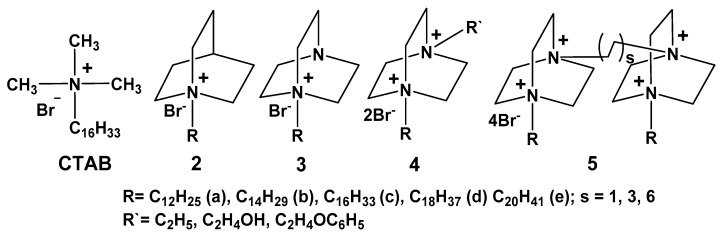
Structural formulas of quaternized derivatives of quinuclidine (**1a**–**d**) and monocationic (1,4-diazobicyclo[2.2.2]octane (DABCO)-n) (**3a**–**d**), dicationic (Et-DABCO-n) (4-Et a–d), (C_2_H_4_OH-DABCO-n) (4-C_2_H_4_OH **a**–**d**), (4-C_2_H_4_OC_6_H_5_ **a**–**d**), and tetracationic gemini n-DABCO-2-Dabco-n (**5a**–**d**) surfactants.

Our group is focused on the development of polyfunctional systems based on the diversity of cationic surfactants. Among them, much attention has been paid to quaternized derivatives of 1,4-diazabicyclo[2.2.2]octane and quinuclidine (Q-Nuc-n). These amphiphilic compounds can be obtained through the simple synthetic route, i.e., the quaternization of DABCO by alkyl bromides. The bicyclic precursor, DABCO, is a commercial product that is widely used as catalyst. Saturated bicyclic scaffold with a single junctional nitrogen (quinuclidine) is common in natural physiologically active substances. This primary bicyclic compound provides many advantages for the design of amphiphilic compounds with diverse structural behavior, involving one to four nitrogen’s bearing alkyl groups of different hydrophobicity [138]. Quaternized derivatives of 1,4-diazabicyclo[2.2.2]octane (DABCO-*n* series) are of special interest, because of their wide applications including their use as antibacterial agents (e.g., [139]) and artificial ribonucleases [140]. Despite the key role of hydrophobicity of surfactants in their micellization and functional activity, this aspect is poorly studied for alkylated DABCO excepting isolated publications. Our work focused on the aggregation activity of mono-(**3**) and di-(**4**) and tetracationic (**5**) derivatives of DABCO (Figure 2) in water, chloroform, and biological fluids [138,141,142], their catalytic and biological activity [142,143], adsorption on the air/water interface [141,144] in both single solution and in the presence of calixarenes [145,146,147], organophosphorus substrates [148,149,150], and polymers [151]. As can be seen from the structural formulas (Figure 2), these surfactant series allow the comparison of the behavior of gemini surfactants bearing two charged fragments and two long-chain alkyl groups with dicationic surfactants bearing one long-chain alkyl fragment.

### 5.1. Aggregation Behavior and Morphology

**Figure 3:**
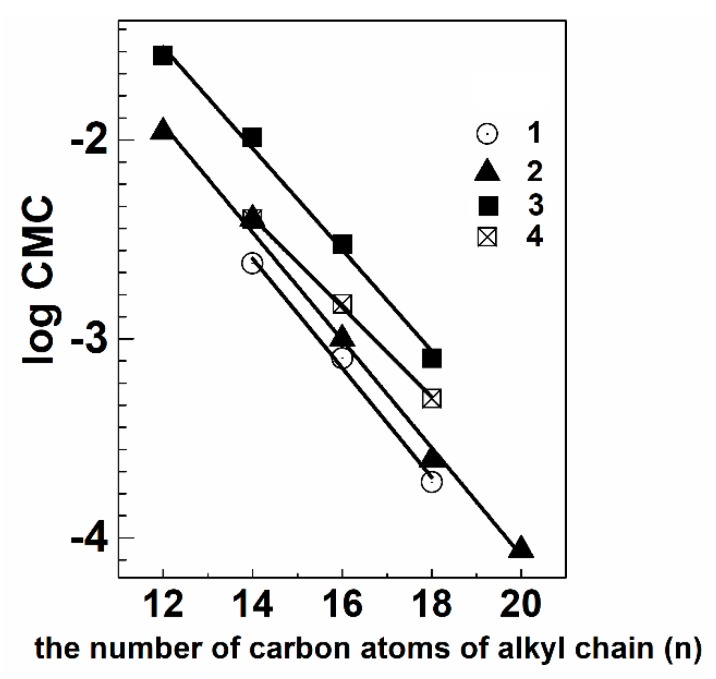
The effect critical micelle concentration (CMC) on chain length (n) and amount of charge of cationic surfactants based on DABCO and quinuclidine, quinuclidine (Q-Nuc-n) (1), mono-DABCO-n (2), di-DABCO-Et-n (3), and tetra n-DABCO-s-DABCO-n (4) 25 °C.

The use of cationic surfactants in the biomedical field requires the assessment of their aggregation parameters (i.e., CMC, aggregation number, Nagg, hydrodynamic radius/diameter of aggregates, R_H_/D_H_, degree of counter-ion binding, and (β)) which are usually determined by different techniques summarized in Table 2. The data demonstrate that CMC values depend both on the hydrophobicity and number of charge fragments of surfactants. An increase in the number of carbon atoms in alkyl groups (n) results in a predictable decrease in CMC in accordance with a linear dependence:

−log *CMC* = (*a* + *bn*) (1)

where *a* and *b* are constants for a for a particular homologous series and temperature. Dicationic single-tailed surfactants, diquaternized derivatives (**4**) show higher CMC as compared to monocationic (**3**), and acyclic (CTAB) analogs (Table 2) (Figure 3). There is a good linear dependence for all Dabco-surfactant series.

−log CMC = 1.25 − 0.28n r^2^ = 0.997 (for Q-Nuc-n) (2)

−log CMC = 1.33 − 0.27n r^2^ = 0.998 (for mono-DABCO-n) (3)

−log CMC = 1.52 − 0.25n r^2^ = 0.997 (for di-DABCO-Et-n) (4)

−log CMC = 0.77 − 0.23n r^2^ = 0.999 (for geminis tetra n-DABCO-s-DABCO-n) (5)

Slopes are decreasing from monocationic DABCO-n (0.27) to dicationic Et-DABCO-n (0.25) and tetracationic n-DABCO-2-DABCO-n (0.23) surfactants. Characteristic slope is 0.28–0.30 for typical ionic surfactants. This behavior is associated with an increase in the number of charged nitrogen atoms and polarity of DABCO-surfactants. The CMC of tetracationic n-DABCO-2-DABCO-n are higher than CMC of monocationic DABCO-n and lower than dicationic Et-DABCO-n with the same alkyl chain. This can be due to the strong electrostatic repulsion of two similarly charged nitrogen atoms, which is not compensated by hydrophobic effect contributed by second alkyl tails as in the case of geminis.

Additives are shown to affect the CMC values. For aqueous **3c** solution the influence of water-soluble calix[4]resorcine [138,145,146], organic electrolytes (sodium salicylate (NaSal), sodium tosylate (NaTos), sodium benzoate (NaBn)), and polyelectrolyte (sodium polystyrene sulfonate, PSS) [152] capable of self-assembling are used. A significant decrease of CMC in the presence of salts was found. The addition of CR groups promotes the formation of mixed aggregates. Recently we have reported about the substantial decrease in CMC of **3c** and **4c** in nutrient broths (Hottinger broth (pH = 7.2) and Sabouraud dextrose broth (pH = 5.6)) up to 60 times [142].

Apart from CMCs, the degree of counter-ion binding β, radiuses of quaternized DABCO derivative aggregates and number of aggregations are determined. For **3a**, **3b**, and **3d**, β ranges from 0.67 to 0.84, while for **3c** it changes from 0.81 to 0.91 [144]. NMR diffusivity data reveal that around the CMC hydrodynamic radiuses equal 15.1, 20.3, and 22.8 Å, while aggregation numbers equal 24, 54, and 72 for **3a**, **3b**, and **3c**, respectively [149,153], i.e., both parameters increase with the hydrophobicity of surfactants. For concentration range above CMC the radiuses and aggregation numbers were shown to depend on the surfactant concentration [154]. At high **3b** concentrations the larger micelles are formed that are characterized by higher numbers of aggregation. This is confirmed by the fluorimetry data indicating that for the **3b** concentrations of 5 × 10^−3^, 7.5 × 10^−3^, and 2.5 × 10^−2^ M aggregation numbers equal 40, 59 and 114, respectively.

**3c** has been shown to belong to amphotropic compounds capable of forming the mesophase of different types, i.e., thermotropic and lyotropic. It has been found that for lyotropic liquid crystals the wider temperature range is observed as compared to thermotropic phase. The existence of lyomesophase within the wide temperature range extends the application of these systems as matrix for the design of nanomaterials and models of membranes in biotechnological protocols. These data are in line with unusual structural behavior of hydrophobized DABCO derivatives highlighted by the dynamic light scattering data. For **3d** ensembles, hydrodynamic diameter has been recorded in the range from 180 to 280 nm and was dependent on the age of the solutions. An increase in the concentration of **3c** was followed by the decrease in the size of aggregates from 180 to 135 nm. The hydrodynamic diameter of **4-Et** particles at concentration around CMC is ca. 110–160 nm. The monitoring of the solutions with time revealed even higher aggregation of particles. The increase in the concentration of **4-Et** was followed by the substantial decrease in the size of aggregates, up to 3–4 nm [141]. Similar decrease of particle size takes place for tetraquaternized derivatives of DABCO. This phenomenon can be due to the change in the morphology of aggregates, e.g., the predominant existence of vesicle structures in the range before and near CMC, with their transfer or organize at the same time more compact micellar form with the growth in surfactant concentration. The fluorescence anisotropy of 1,6-diphenyl-1,3,5-hexatriene in **4b** solutions supports our suggestions on the change of the morphology of aggregates.

### 5.2. Solubilization and Controlled Binding/Release of Hydrophobic Guests

**Figure 4:**
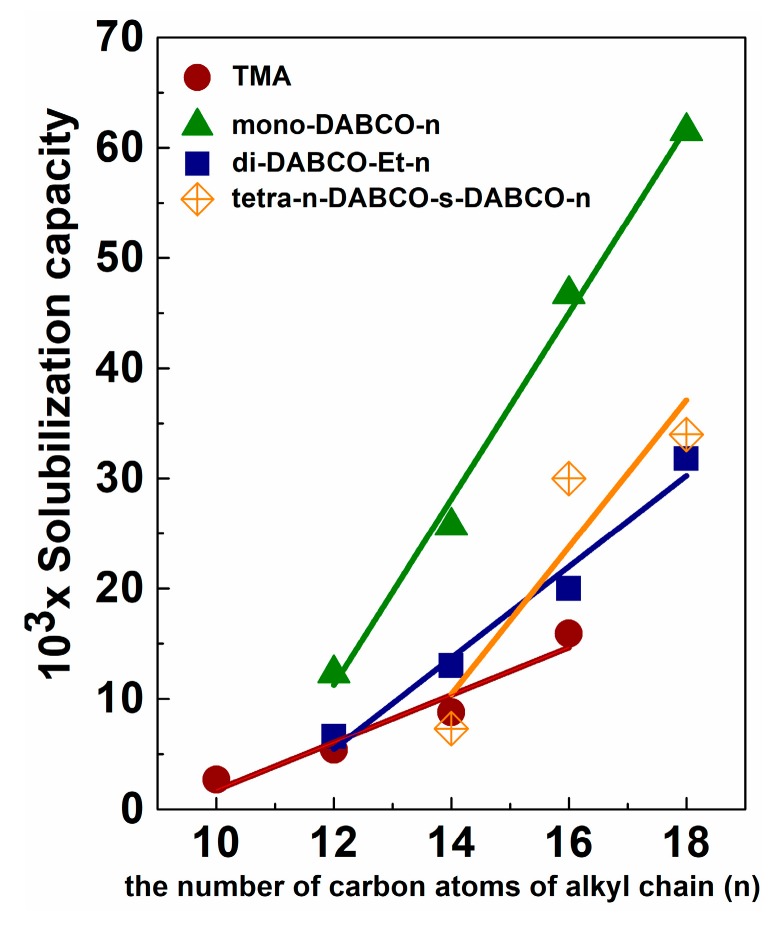
The solubilization capacity of micellar aggregates based on quaternized derivatives of DABCO and trimethyl ammonium (TMA) analogs toward the dye Orange OT.

Solubilization capacity of aggregates is a key property responsible for the use of cationic surfactants as nanocarriers. This stimulates the search for novel effective formulations for solubilization of hydrophobic drugs and diagnostic probes, and therefore the elucidation of structure-activity relation is a challenging task. While the factor of hydrophobicity of surfactants in terms of alkyl tail length is discussed in literature, the influence of structure of head groups is less studied and understood [155]. In our work [156], the higher solubilization capacity toward hydrophobic dye Orange OT is revealed for quaternized DABCO as compared to trimethyl ammonium analogs (Figure 4). 

Solubility of Orange OT linearly increases with alkyl chain length of surfactants and follows the relations:

S = −0.0197 + 0.00215n, r = 0.997 (for TMA)(6)

S = −0.0899 + 0.00843n, r = 0.997 (for mono-DABCO-n) (7)

S = −0.0442 + 0.00413n, r = 0.997 (for di-DABCO-Et-n) (8)

S = −0.08303 + 0.0066n, r = 0.97 (for geminis tetra n-DABCO-s-DABCO-n) (9)

This may be explained by the fact that regardless of the similarity of CMC values for trimethyl ammonium-n and DABCO-n series, their structural behavior differs markedly. Moreover, DABCO based cationic surfactants exhibit superior solubilization properties over cationic surfactants with triphenylphosphonium head groups [155], although the latter are characterized by advanced micellization activity, i.e., their CMCs are ca. 10-fold lower compared to that of DABCO-series. Different effect of the hydrophobicity factor occurs in the case of trimethyl ammonium and DABCO series. An increase in alkyl chain length of surfactants exerts much more marked effect on the solubilization capacity of DABCO surfactant as compared to trimethyl ammonium series.

The decrease in solubilization capability of di-CS-n with the same alkyl chain may result from their lower aggregation numbers and lower packaging in di-CS-n micelles.

Many advantages in the development of effective drug delivery systems may be obtained upon the design of mixed assemblies. Nowadays, the interest in mixed compositions has markedly increased due to the development of novel direction of supramolecular chemistry focusing on the formation of supramolecular amphiphiles [159]. Growing interest in supramolecular amphiphiles is determined by two aspects [160]: (i) easy design of supramolecular amphiphiles that can be formed through various non-covalent interactions (hydrogen bonds, metal–ligand coordination, electrostatic and π-stacking, as well as host–guest interactions) [161] and (ii) dynamic character of non-covalent associates that enable easy control of the properties of formed structures [162]. The latter is essential for the design of smart supramolecular materials, which are responsive to various factors (e.g., pH, temperature, light, magnetic field, and oxidation) [163]. The application of mixed surfactant-calixarene systems opens additional opportunities in this field, since the involvement of macrocyclic receptors may provide a high selectivity of the guest binding based on principles of the molecular recognition. It is known that macrocycles can take part in guest–host interactions due to the presence of molecular cavity and preorganization of functional groups [164]. A key point is that the self-assembling mechanism of surfactants and calixarenes may be strongly different that provides the possibility for the development of nanocarriers with controlled binding/release properties by means of inducing the morphological rearrangements. Besides, specific geometry of calixarene molecules emphasize their potential as drug delivery systems [165].

**Figure 5:**
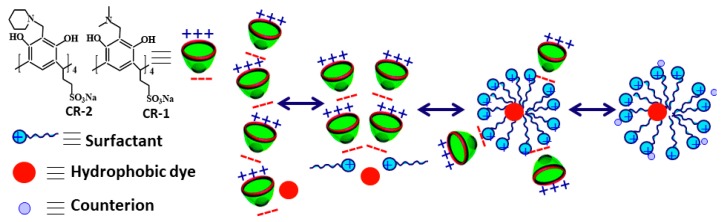
Changes in the supramolecular architecture with the variation in the calixresorcine ratio: The controllable guest release.

As mention above in the presence of CR-1, cationic surfactant **3c** starts to associate at the lower concentration and undergoes further morphological transitions with an increase in the concentration [145]. The solubilization study with the use of hydrophobic dye Orange OT demonstrated that only mixed aggregates enriched by **3c** are capable of binding the organic probe, while mixed system where the surfactant is a minor component shows no binding capacity towards Orange OT (Figure 5). This finding can be used for the design of nanocarriers with controllable binding-release properties.

The packing mode, morphology, and size of assemblies and their solubilizing properties depend on the structure of cationic surfactants, in particular, their charge character and the ratio of the components in mixed system. The key role of electrostatic interactions in the formation and rearrangement of mixed supramolecular structures has been demonstrated by Pashirova et al. [138]. It was shown that the systems with either the minor or predominant content of cationic surfactants exhibit the lowest and the highest solubilizing capacity, respectively, toward hydrophobic organic guest. This is essential from the viewpoint of the design of nanocarriers with controlled and reversible binding capacity toward therapeutic agents or biomolecules.

**Figure 6:**
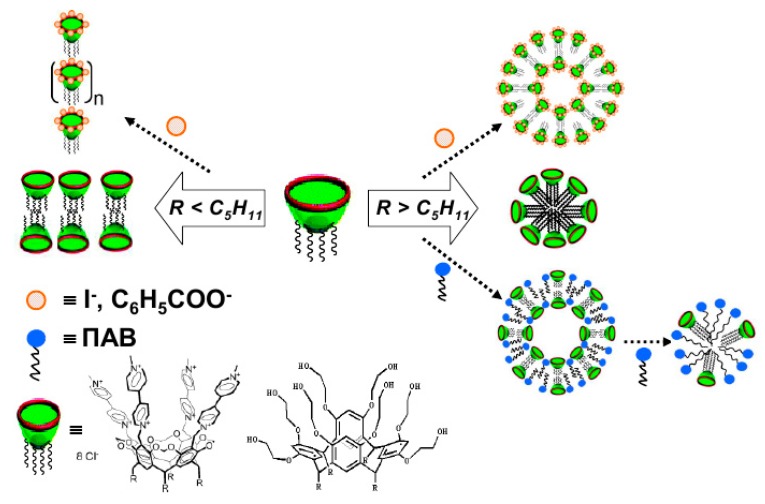
Changes in the morphology with the variation in the surfactants-calixresorcine ratio.

The factor of hydrophobicity is differently displayed in the case of macrocyclic amphiphiles, i.e., calixarenes compared to conventional surfactants [166]. Unlike typical surfactants, the influence of alkyl chain length on the size and morphology of aggregates, their surface and functional activity have critical character, in particular, the change in the association model occurs in the case of R = C_5_H_11_ (Figure 6). This finding is of key importance from the viewpoint of development of smart nanocarriers based on mixture of surfactant and macrocycles. The idea of modification of the surfactant-calixarene nanocarriers can be improved by the knowledge of other structural factors controlling the morphological behavior and solubilization activity of superamphiphilic compositions. The modulation of properties can be achieved by the variation of the nature of dispersion media, component ratio, and even the nature of counter-ions [166,167].

The data obtained allow anticipating that novel DABCO-based cationic surfactants along with geminis may have promising structural units for the design of nanocarriers for gene and drug delivery.

### 5.3. Supramolecular Catalysis

One of the challenges in the modern catalysis is the development of artificial catalytic systems modelling the elementary mechanisms of enzyme catalysis, which requires the elucidation of factors controlling the rate enhancement in living systems. Supramolecular catalysts are assumed to exhibit biomimetic character, since their mechanism includes the formation of the substrate-catalyst complexes [168], which further undergo chemical transformation, with both stages contributing to the change rate. The same mechanism involving the preliminary binding the substrate occurs in enzyme catalysis. Therefore, the study of highly effective and selective supramolecular catalytic systems may shed light on the factors controlling high efficacy and substrate specificity of biocatalysts. The knowledge of these factors and their reproducing in technological protocols are of key importance from the viewpoint of criteria of green chemistry, since they make it possible to decrease the concentrations of catalysts in a marked extent and hence to fabricate ecologically friendly systems [169].

At present, the main lines of improvement of micellar catalysts are enhancing the efficiency and selectivity of catalytic systems and reducing the surfactant concentration. This can be achieved by varying the surfactant structure, including the nature of the head group, and by passing to dimeric (gemini) surfactants. Catalytic activity of the systems based on quaternized derivatives of DABCO is exemplified by basic hydrolysis (in water) and aminolysis (in chloroform) of phosphorus acid esters (Figure 6) monitored by methods of spectrophotometry [147,148,149,150] and NMR spectroscopy [148]. These reactions may serve as convenient model for studying of the most important biochemical reactions. Moreover, the cleavage of phosphoester bonds is one way of detoxification for organophosphorus neurotoxins and toxicants [2].

**Figure 7:**
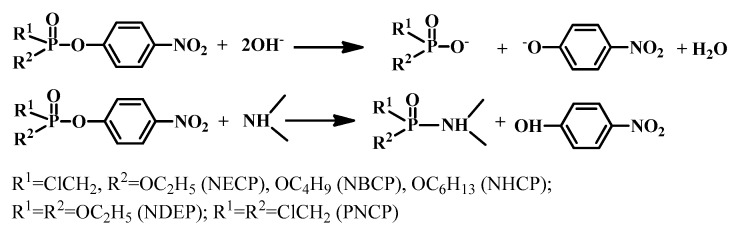
Scheme of alkaline hydrolysis of phosphorus acid esters.

Our studies focusing on the evaluation of the structure-activity relations revealed that the cationic surfactants catalyze the hydrolysis of NBCP (Figure 7). Their catalytic activity depends on the structure of the head group of the surfactant and increases in the order CTAB ≈ morpholinium surfactant < cetylpyridinium bromide < monoquaternized DABCO derivatives. The catalytic effect of aggregates of DABCO-surfactants increases with the increase of their hydrophobicity and with the decrease of the alkalinity of the solution and can achieve ca. 200 times [149]. Chemical reactions in organized solutions containing metallomicelles formed by complexes of metals with the surfactant-based ligand or involving aggregates solubilizing metallocomplexes with no hydrophobic long chain fragment are of special importance. This is due to the fact that such kind of assemblies can show enhanced functional activity including catalytic effect, which provides further possibility for the design of new materials, destruction of poises and ecotoxicants, and development of biomimetic technological approaches. Investigations of the effect of DABCO-surfactant/lanthanum nitrate systems on basic hydrolysis of NECP, NPCP, and NHCP revealed the high catalytic effect of more than two orders of magnitude [157]. A system based on aminomethylated calix[4]resorcinarene (CR-1) DABCO surfactant (**3c**) and their mixtures has shown the catalytic effect on the hydrolysis of phosphorus acid esters. It was established that the presence of La(NO_3_)_2_ leads to a 650-fold increase in the catalytic effect [147]. The presence of La(III) ions enhanced the catalytic effect of calixarene aggregates due to complex manifestations of micellar and homogeneous (electrophilic) catalyst, and to favorable changes in the characteristics (the degree of binding of counter-ions and surface charge). The favorable effect of amphiphilic additives is due to their ability to form individual and mixed aggregates in aqueous solutions, the transition of reagent from the bulk solution into these aggregates, and the turning the chemical process to the new direction.

### 5.4. Antimicrobial Activity

In addition to the aggregation and catalytic properties, antimicrobial activity of quaternized DABCO derivatives has also been studied, whereby the correlation of antimicrobial and aggregation properties is analyzed [148]. The bacteriostatic activity is decreased with increasing the alkyl chain length for DABCO-n from dodecyl to its octadecyl analogue. DABCO-16 and DABCO-18 exhibit a highest bacteriostatic activity (MIC = 0.3, 1.9, and 6.3 µg/mL against *St. aureus*, *B. cereus,* and *E. coli*, respectively). Generally, bacterial strains were the most sensitive toward tetradecylrimethylammonium bromide in series of n-alkyltrimethylammonium bromides [170] and toward C14 (for gram-positive bacteria), and C16 (for gram-negative bacteria) in series of alkyldimethylbenzylammonium chlorides [171]. The influence of head group is mostly evident in the case of *St. Aureus*, for which the transition from CTAB to quaternized quinuclidine and monoquaternized DABCO derivatives provide a tenfold increase in bactericidal activity. Much higher antifungal activity exemplified by *Tr. gipseum* and *C. albicans* should also be noted for **3d** as compared to CTAB. Fungistatic activity of **3d** toward these strains is by two-fold and four-fold higher and fungicidal activity is by 16-fold and 10-fold higher than those for CTAB. The bactericidal and fungicidal activity of Q-Nuc-18 is 1.95 μg·mL^−1^. This value is much higher than for its DABCO-n analogues. Q-Nuc-18 has bactericidal and fungicidal activity two times (against *St. aureus*) and eight times (against *B. cereus*) higher than antibiotics Norfloxacin and antifungal Ketoconazole. Q-Nuc-16 has the highest bactericidal activity. It is six times (against *St. aureus*) and 15 times (against *B. cereus*) higher than the bactericidal activity value of Norfloxacin [143].

Second charge moiety exerts a negative influence on antifungal activity, e.g., the transition from monoquaternized derivatives of DABCO (**3c**) to diquaternized derivatives of DABCO (**4c-Et**) results in four-fold decrease in antifungal activity toward *Tr. gipseum* and in ten-fold decrease toward *C. albicans.* At the same time, antimicrobial activity toward *B. Cereus*, *E. coli,* and *St. Aureus* remains practically the same. Data obtained demonstrate that the introduction of bicyclic polar fragment into surfactant platform may be of interest from the viewpoint of the design of antimicrobial preparations (in particular, antifungal ones) of little toxicity. The highest effect can be achieved by the increase of alkyl chain length and optimization of hydrophilic-lipophilic properties [148]. In case of tetracationic surfactants, 12-Dabco-2-Dabco-12 is the most active without hemolytic activity at concentration 3.1 μg·mL^−1^. The MICs of 2-Dabco-2-Dabco-12 are 3.9, 7.8, and 31.3 µg/mL against *St. aureus*, *B. cereus,* and *E. coli*, respectively. Likely this is due to a different mechanism of action of these surfactants. Four charged nitrogen atoms and two alkyl chains of n-Dabco-2-Dabco-n may accommodate faster interaction and penetration of DABCO-surfactants through the bacterial cytoplasmic membrane. These results clearly indicate that the antibacterial activity of DABCO-surfactants is strongly affected by their structure (alkyl chain length and the amount of charged nitrogen).

The ways of decreasing the toxicity of biologically active compositions with the preservation of their useful properties is an important task. For this purpose, the effect of N-methyl-D-glucamine additive on the antimicrobial activity of monoquaternized derivatives of DABCO and its aggregation and solubilization parameters has been assessed [142]. Although N-methyl-D-glucamine itself shows no biological activity, its addition to DABCO surfactants enables one to decrease the concentration of the active ingredient (DABCO surfactants) in antimicrobial composition by two times thereby reducing the toxicity of cationic surfactant on human red blood cells. This provided a remarkable increase in solubilization capacity of DABCO surfactants aggregates (by ~4 times) [142]. Thus, DABCO cationic surfactants are able of forming micellar aggregates in water with high solubilization capacity for hydrophobic dyes, drugs and organophosphorus toxicants. In these systems, the strategy can be realized for controlled binding/release of hydrophobic probes by means of the change in morphology of mixed aggregates and hence their affinity toward hydrophobic guests. They demonstrate advanced antimicrobial activity as compared to analogs with acyclic head groups. This anticipates that alkylated DABCO may be promising candidates for application in biotechnologies, which can be used for the development of nanocarriers with polyfunctional activity.

## 6. Conclusions

The survey of the recent publications strongly supports cationic amphiphiles as a very promising material in modern nano- and biotechnologies. The development of innovative functional nanocarriers based on cationic surfactants allows understanding the structure-activity correlation between chemical structure of the amphiphiles molecules and their aggregation behavior and functional activity (e.g., as drug delivery systems, catalysts, sensors). Noteworthy, the selection of papers for this review judged from the idea to reflect key trends in the field of bio- and nanotechnological applications of amphiphile-based systems, especially those positively charged. As can be seen, these trends focus on the development of drug delivery systems answering the criteria of green chemistry and biomimetic design. Therefore, the majority of strategies are aimed at the fabrication of soft and smart nanosized constructions, with the minimization of concentration of building blocks and their side effect attained. Moreover, it is still challenging to prepare nanocarriers with acceptable toxicological profile. In future studies, research towards the optimization of the formulations, and the study of structure-properties correlations are strongly needed, which brings to the forefront the systematic studies involving homological series of amphiphilic compounds and their directed functionalization. A steady trend that is assumed to be preserved consists in the use of gemini and polycationic compounds allowing for markedly diminish the usage of self-assemble units due to their low aggregation threshold. The same is true for the very promising compounds bearing cleavable moiety, e.g., ester and amide groups. Meanwhile, biomimetic approach assumes the wider use of natural components; therefore, amphiphiles bearing biofragments remain of special interest. Separate line in nanomedicine strategy involves the use of amphiphilic peptides and peptide-conjugated formulations for delivery of both hydrophilic and hydrophobic therapeutic molecules. Noteworthy that preparation of drug delivery systems involving biological units may be administered through both covalent and non-covalent protocols. While the former provides more advantages in functional activity, the latter is less expensive and assumes the simpler and faster experiments. Therefore, the parity is probably expected to be preserved between these strategies. To sum up, one of the most relevant applications of cationic surfactants is the design of modified nanocontainers for drug and gene delivery. However, more in-depth studies need to be done, such as in vivo experiments to evaluate functional activity of the loaded cargos, due to the achievement of synergetic structural behavior and multidrug delivery with stimuli responsive activity.

## Figures and Tables

**Table 1:** Examples of amino-acid residue containing quaternary ammonium compounds (QACs).

Amino Acid	Type of QAS	Type of Aggregates	References
Alanine	Gemini ester quat surfactants	/	[53]
Arginine	Alkylated Arg (LAM), gemini alkylated Arg (C_6_(LA_2_); C_9_(LA_2_), C_12_(LA_2_))	LAM, C_6_(LA_2_) -Micelles, C_9_(LA_2_), C_12_(LA_2_)-vesicles	[72]
	Diacylglycero Arg	Vesicles	[70]
Lysine	/	Lysine-surfactants in liposomes	[75,76]
		Lysine-gel based systems	[77,78]
Serine	/	Serine-gene delivery systems	[48,73]

**Table 2:** Values of CMC of cationic surfactants in water.

Surfactant	Additives	CMC × 10^3^ (M) (Based on Different Techniques)						Refs
		Tensiometry	Conductometry	Potentiometry	NMR	Fluorimetry	Spectrophotometry	
2b		2.5	2.85			3.0	1.5	
2c		0.85	0.6			0.94	0.8	
2d		0.2	0.24			0.3	0.3	
3a		11	14	16	15	-		[144,149]
3b		4.0	3.0	3.7	3.4	4.3		[144]
3c		1.0	1.0	1.9	0.85			[144,153]
3d		0.24	0.11	0.22	0.11	-		[144,149]
4a-Et		26.5	28.4	29.7			28	[157]
4b-Et		10.3	8.5	8.4		8.1	9.5	[157]
4c-Et		3.0	3.1	2.0	-	-	2.3	[157]
4c-EtOH		2.0	2.5	3.0	-	-	-	[138,157]
4d-Et		0.80	0.83	0.98			1.1	[157]
5b		4.0	3.0	2.0		4.2	3.6	[158]
5c		1.5	1.7	2.3		0.8	1.7	[158]
5d		0.5	0.5	0.15		0.29	0.2	[158]
3c	CR-1	2	0.1	-	-	-	-	[138]
4c-Et	CR-1	1.5	1.5	-	-	-	0.8	[138]
5c	CR-1	1	0.4	-	-	-	-	[138]
3c	CR-1	0.4	0.1	4.9	0.45	-	5.0	[145]
